# Supplementary material for: Comparative Evaluation of the Antiglycation and Anti-α-Glucosidase Activities of Baicalein, Baicalin (Baicalein 7-O-Glucuronide) and the Antidiabetic Drug Metformin
Source: Pharmaceutics. 2022 Oct 9;14(10):2141. doi: 10.3390/pharmaceutics14102141 (PMC9612222; doi:10.3390/pharmaceutics14102141)
Supplement: Supplementary file 1 [file pharmaceutics-14-02141-s001.zip › pharmaceutics-1841170-supplementary.pdf]

Supplementary Materials: Comparative Evaluation of the Anti-glycation and Anti-α-Glucosidase Activities of Baicalein, Baicalin (Baicalein 7-O-Glucuronide) and the Antidiabetic Drug Metformin

Guglielmina Froldi*, Francine Medjiofack Djeujo, Nadia Bulf, Emma Caparelli and Eugenio Ragazzi

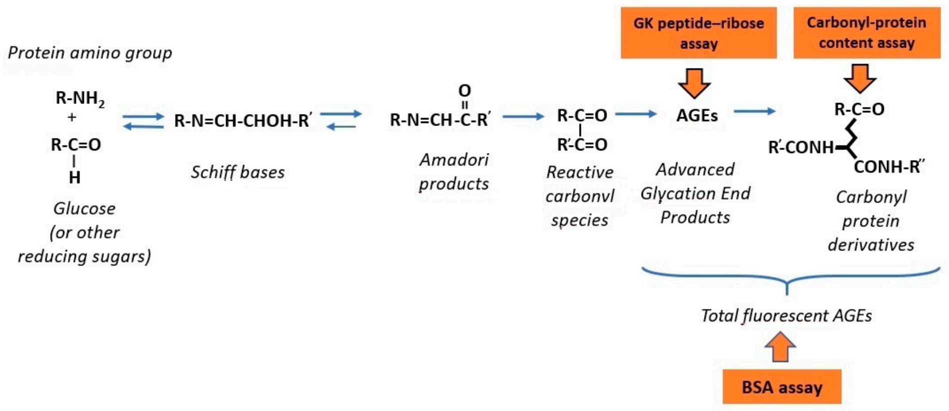

Figure S1. Scheme of AGE formation and protein carbonylation.

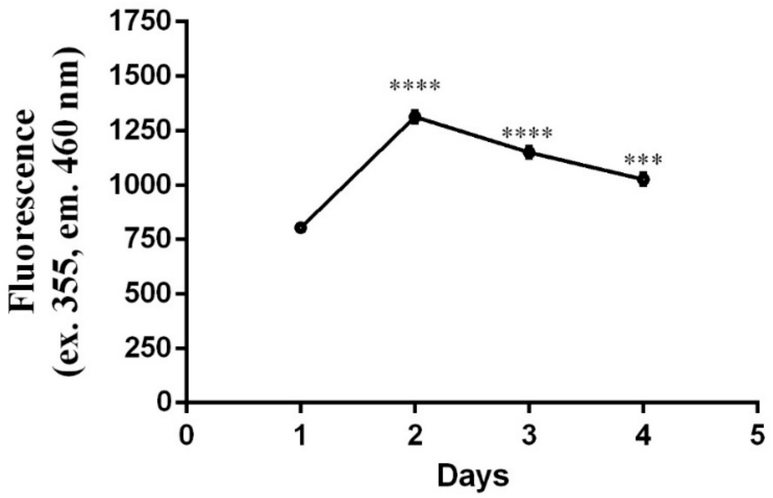

Figure S2. GK–peptide kinetic curve obtained by measuring the fluorescence intensity at 355 nm excitation and 460 nm emission wavelengths over several days of incubation with 0.1 M ribose. Data are reported as mean ± SEM of 3–6 experiments.

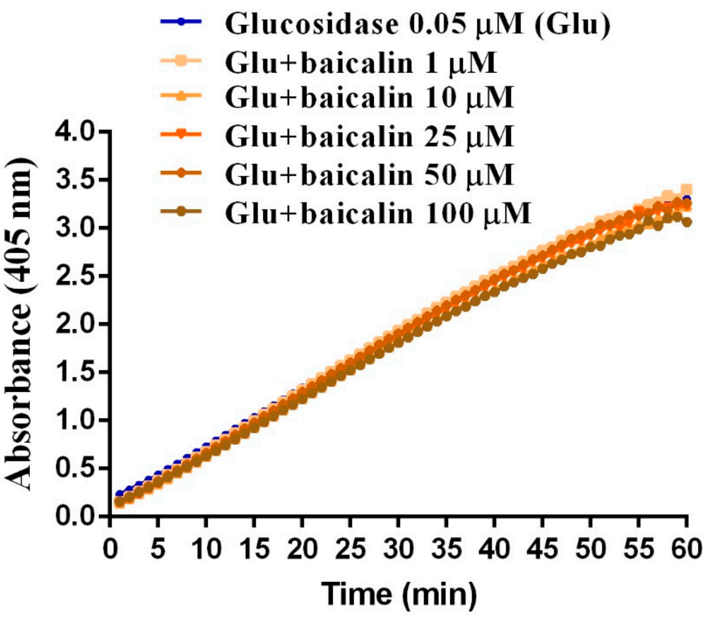

Figure S3. An example of kinetic curves of 0.05 μM α–glucosidase and 2 mM *p*NPG in the presence of increasing concentrations of baicalin.

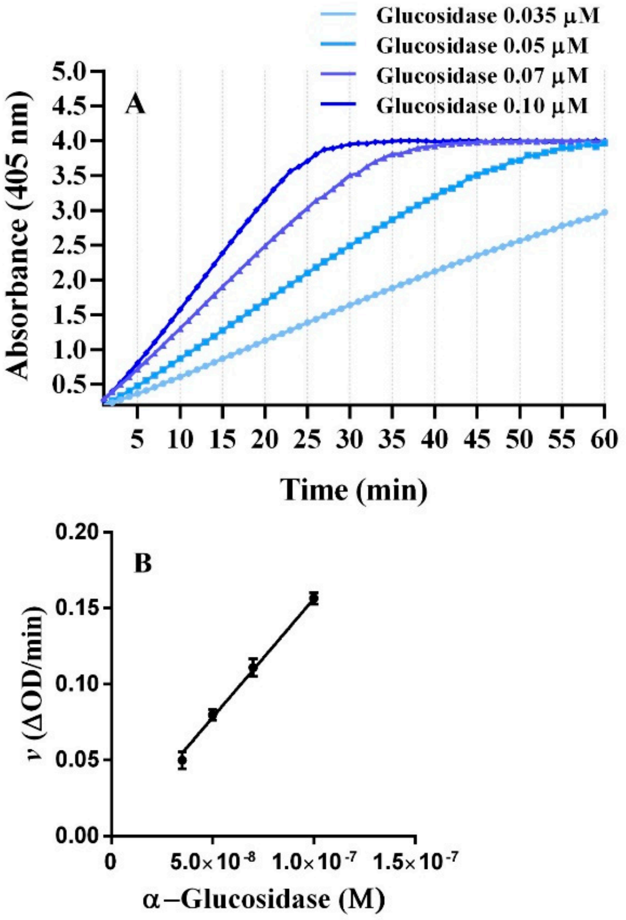

Figure S4. Enzyme kinetics observed with different concentrations of α–glucosidase and 2 mM *p*NPG (**A**). Graph "v versus [α–glucosidase]" (**B**).

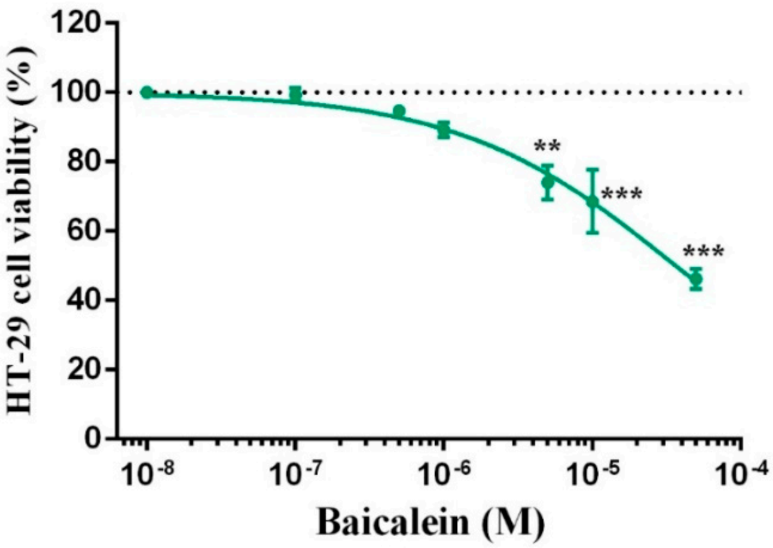

Figure S5. Effects of baicalein on the viability of human colon adenocarcinoma (HT-29) cells. ** $p < 0.01$ and *** $p < 0.0001$ vs control (cell viability without baicalein).
